# Editorial: State-of-the-art artificial intelligence methods in neurodegeneration

**DOI:** 10.3389/fneur.2022.1112639

**Published:** 2023-01-23

**Authors:** Iman Beheshti, Daichi Sone, Zhijun Yao, Norihide Maikusa

**Affiliations:** ^1^Department of Human Anatomy and Cell Science, University of Manitoba, Winnipeg, MB, Canada; ^2^Department of Psychiatry, Jikei University School of Medicine, Tokyo, Japan; ^3^Gansu Provincial Key Laboratory of Wearable Computing, School of Information Science and Engineering, Lanzhou University, Lanzhou, China; ^4^Center for Evolutionary Cognitive Sciences, Graduate School of Art and Sciences, The University of Tokyo, Tokyo, Japan

**Keywords:** algorithms, neurodegeneration, neuroimaging, computational intelligence, data fusion

In the last decade, artificial intelligence methods have been widely used in neurodegeneration diseases. Artificial intelligence methods, particularly machine-learning algorithms, would allow radiologists, scientists, and clinicians to address for early diagnostic tools (1), predict longitudinal brain changes (2), and effective treatments for patients who suffer from different types of neurodegenerative disorders (3). In this Research Topic, we aimed to present the state-of-the-art and novel algorithms, methodologies, and applications of artificial intelligence methods in neurodegenerative disorders. We focused on advanced artificial intelligence methods for applications in Alzheimer's disease (AD), Parkinson's disease (PD), motor neuron diseases, Huntington's disease, frontotemporal lobar degeneration, and other neurological disorders to advance our understanding of these diseases using artificial intelligence models. In total, we received eight original research papers in this Research Topic; five of them (written by 35 authors across the globe) were chosen for publication after peer reviews. All of the accepted articles focused on developing new artificial intelligence methods in the area of neurodegenerative disorders or elderly people. In this editorial, we summarize the main findings and viewpoints presented in each of the accepted articles.

In a paper of this Research Topic entitled “*Automatic Classification and Severity Estimation of Ataxia From Finger Tapping Videos*,” Nunes et al. demonstrated the reliability of finger-tapping videos for classifying Ataxia subjects from participants with Parkinsonism patients and from healthy controls. In addition, the authors aimed to predict overall disease severity based on finger-tapping video data and machine-learning models. To this end, they extracted time series features from video recordings of a finger-tapping task. They used logistic regression and ridge regression algorithms for classification and regression tasks, respectively. They reported an AUC of 0.92, 0.91, and 0.68 for distinguishing ataxia from healthy control, ataxia from Parkinsonism, and Parkinsonism from healthy controls, respectively. In addition, a correlation coefficient >0.64 was reported between predicted and actual clinical scores (i.e., Total BARS for ataxia and UPDRS for Parkinsonism subjects).

In the article “*Temporal Progression Patterns of Brain Atrophy in Corticobasal Syndrome and Progressive Supranuclear Palsy Revealed by Subtype and Stage Inference (SuStaIn)*” (Saito et al.) explored the difference in the temporal progression patterns of brain atrophy between Corticobasal degeneration (CBD) and progressive supranuclear palsy with Richardson's syndrome (PSP-RS) patients using anatomical MRI data coupled with a novel unsupervised machine learning technique called SuStaIn. The proposed SuSta technique was applied to the brain features of all subjects to discover the disease subtypes and trajectories of CBS and PSP-RS. Using this technique, the authors discovered different temporal progression patterns of brain atrophy between CBS and PSP-RS groups which was able to distinguish two groups with an accuracy of 87.5%, indicating that SuStaIn can be viewed as a reliable method for separating PSP-RS patients from CBS patients and predicting treatment options among them.

In another article, entitled “*Application of High-Frequency Conductivity Map Using MRI to Evaluate It in the Brain of Alzheimer's Disease Patients*,” the authors aimed to explore the differences among three participant groups of cognitively normal (CN) elderly people, amnestic mild cognitive impairment (MCI) and AD in terms of a high-frequency conductivity (HFC) mapping technique extracted from a 3T MRI system. They also investigated the association between HFC values and cognitive decline as well as the utility of the HFC technique for classifying AD patients from CN (Park et al.). They reported a higher HFC value in the AD group in comparison to the CN and MCI groups. Besides, they stated a positive association between HFC values and age as well as between HFC values and disease severity in AD. Using the HFC value in the insula, the authors achieved to an AUC of 0.902 for distinguishing AD patients from CN which was higher than gray matter volume (GMV) data in the hippocampus (AUC = 0.880). The authors pointed out that this classification between AD patients from CN can be improved by concatenating the insular HFC value with hippocampal GMV to an AUC of AUC = 0.928.

In a paper titled “*Identifying Depression in Parkinson's Disease by Using Combined Diffusion Tensor Imaging and Support Vector Machine*,” Yang et al. used diffusion tensor imaging (DTI) with a support vector machine algorithm to identify depressed PD patients. To this end, they extracted fractional anisotropy (FA) and mean diffusivity (MD) among depressed PD patients, non-depressed PD, and healthy control subjects. In the analysis stage, they stated an increased MD signal in the left superior longitudinal fasciculus-temporal and right cingulum (cingulate gyrus) as well as decreased FA signal in the right cingulum (cingulate gyrus), bilateral inferior longitudinal fasciculus, bilateral corticospinal tract, left cingulum hippocampus, and bilateral superior longitudinal fasciculus among depressed PD patients compared to non-depressed PD patients. The Support Vector Machine algorithm showed an accuracy of 0.73 for distinguishing depressed PD patients from non-depressed PD patients with DTI data. They concluded that depression in PD is linked to changes in the white matter's microstructure and machine learning models with DTI data could be valuable for detecting depression in PD.

In the last paper in this Research Topic entitled “*Automated Evaluation of Conventional Clock-Drawing Test Using Deep Neural Network: Potential as a Mass Screening Tool to Detect Individuals With Cognitive Decline*,” Sato et al. aimed to develop an automated cognitive decline assessment based on Clock-Drawing Test (CDT) data and deep neural network model. This study was conducted on a large set of CDT images (*N* = 40,000) from the National Health and Aging Trends Study (NHATS) database. The authors reported a good balance accuracy of 90% for identifying elderly people with cognitive decline from those without, indicating that the proposed method can be used as a robust screening tool for identifying dementia or signs of impaired executive function among that group of subjects.

In conclusion, these five accepted papers included in this Research Topic provided unique perspectives on addressing clinical questions using advanced artificial intelligence methods in the area of neurodegeneration.

## Author contributions

All authors listed have made a substantial, direct, and intellectual contribution to the work and approved it for publication.
